# Retraction: Inhibition of RIP1-RIP3-mediated necroptosis attenuates renal fibrosis via Wnt3α/β-catenin/GSK-3β signaling in unilateral ureteral obstruction

**DOI:** 10.1371/journal.pone.0340046

**Published:** 2026-01-07

**Authors:** The PLOS One Editors

After this article [1] was published, concerns were raised about Figs 1, 2, 4-7.

Specifically:

- The following western blot panels appear similar when the aspect ratio is adjusted:
  - The Fig 1C p-RIP1 panel*, the Fig 1D p-MLKL panel*, and the Fig 4A NOX-4 panel* of this article [1], and the Fig 1E RIP3 panel of [2].
  - The Fig 1D P-RIP3 panel and the Fig5B Parkin panel.
  - The Fig 2APro-IL-18 panel* and the Fig 6C Bax panel* of this article [1], and the Fig 5C Parkin panel of [2].
  - The Fig 2A pro-IL-1β panel* of this article [1] and the Fig 5C PINK1 panel of [2].
  - The Fig 2B Pro-IL-18 panel and the Fig 7A Wnt3a panel.
  - The Fig 4A MnSOD panel* of this article [1] and the Fig 3B Bcl-2 panel of [2].
  - The Figs 5B and Fig 7A β-actin panels* of this article [1], and the Fig 4C β-actin panel of [2].
  - The Fig 6C β-actin panel* of this article [1] and the Fig 7B β-actin panel of [2].
- The following histology staining results appear to fully or partially overlap:
  - The Fig 1A Sham panel* of this article [1] and the Fig 1B Sham panel of [2].
  - The Fig 3B Sham panel* of this article [1] and the Fig 3A Sham panel of [2].
- The following immunoblotting results appear similar:
  - The Fig 1B UUO+ICG-001 panel* of this article [1] and the Fig 1C UUO+Dapa panel of [2]
- The following electron microscopy results appear similar:
  - The Fig 3C UUO+GSK872 panel of this article [1] and the Fig 2B UUO+LCZ panel in [3, retracted in 4].
- The following TUNEL assay results appear to partially overlap:
  - The Fig 6B UUO+GSK872 and UOO+ICG-001 panels.
  - The Fig 6B UUO+Nec-1 panel* of this article [1] and the Fig 6A UUO+Dapa panel of [2].
- The following FACS results appear similar:
  - The Fig 6D H2O2+ICG-001 panel* of this article [1] and the Fig 6C H2O2+Dapa panel of [2].

Additionally, PLOS noted that the study may not be compliant with PLOS’ Animal research policy. Specifically, the ethics approval number reported in article [1] postdates the ethics document provided at submission. The inconsistency between the approval number and the submitted documentation raises concerns that the study may not have had valid prior approval.

The authors did not respond to queries about these concerns. In the absence of underlying data, the above issues remain unresolved.

In light of the above concerns, which question the reliability and integrity of reported results and conclusions, the *PLOS One* Editors retract this article.

All authors either did not respond directly or could not be reached.

The above panels flagged with an * report material that is similar to that published in [2], which was published in 2022 by Frontiers under a CC BY 4.0 license by a different author group [2]. The original article was not attributed in [1]. Therefore, the above panels flagged with an * are subject to the license that applies to the original article. Please provide due attribution to the original publication when referring to this content.
